# Dietary Patterns and Risk Factors of Frailty in Lebanese Older Adults

**DOI:** 10.3390/nu13072188

**Published:** 2021-06-25

**Authors:** Nathalie Yaghi, Cesar Yaghi, Marianne Abifadel, Christa Boulos, Catherine Feart

**Affiliations:** 1 Department of Nutrition & Dietetics, Faculty of Pharmacy, Saint Joseph University of Beirut, P.O. Box 17-5208 Mar Mikhael, Beirut 1104 2020, Lebanon; nathalie.yaghi@usj.edu.lb (N.Y.); christa.boulos@usj.edu.lb (C.B.); 2 Department of Gastroenterology, Faculty of Medicine, Saint Joseph University of Beirut, P.O. Box 17-5208 Mar Mikhael, Beirut 1104 2020, Lebanon; cesar.yaghi@usj.edu.lb; 3 Hotel-Dieu de France of Beirut University Hospital, P.O. Box 166830, Alfred Naccache Blvd, Beirut, Lebanon; 4 Laboratory of Biochemistry and Molecular Therapeutics, Faculty of Pharmacy, Pôle Technologie-Santé, Saint Joseph University of Beirut, P.O. Box 17-5208 Mar Mikhael, Beirut 1104 2020, Lebanon; marianne.abifadel@usj.edu.lb; 5 LEHA team, INSERM U1219, Université de Bordeaux, F-33000 Bordeaux, France

**Keywords:** frailty, dietary pattern, malnutrition, food groups, Mediterranean dietary pattern, Westernized dietary pattern, older adults, cross-sectional study

## Abstract

Factors associated with frailty, particularly dietary patterns, are not fully understood in Mediterranean countries. This study aimed to investigate the association of data-driven dietary patterns with frailty prevalence in older Lebanese adults. We conducted a cross-sectional national study that included 352 participants above 60 years of age. Sociodemographic and health-related data were collected. Food frequency questionnaires were used to elaborate dietary patterns via the K-mean cluster analysis method. Frailty that accounted for 15% of the sample was twice as much in women (20%) than men (10%). Identified dietary patterns included a Westernized-type dietary pattern (WDP), a high intake/Mediterranean-type dietary pattern (HI-MEDDP), and a moderate intake/Mediterranean-type dietary pattern (MOD-MEDDP). In the multivariate analysis, age, waist to height ratio, polypharmacy, age-related conditions, and WDP were independently associated with frailty. In comparison to MOD-MEDDP, and after adjusting for covariates, adopting a WDP was strongly associated with a higher frailty prevalence in men (OR = 6.63, 95% (CI) (1.82–24.21) and in women (OR = 11.54, 95% (CI) (2.02–65.85). In conclusion, MOD-MEDDP was associated with the least prevalence of frailty, and WDP had the strongest association with frailty in this sample. In the Mediterranean sample, a diet far from the traditional one appears as the key deleterious determinant of frailty.

## 1. Introduction

The frailty phenotype is a multifactorial syndrome associated with aging, characterized by unintentional weight loss, self-reported exhaustion, muscular weakness, slow walking speed, and low physical activity [1]. Physical frailty is also recognized as a risk factor for mortality, increased morbidity [2], malnutrition [2], and falls [3,4]. If not managed, frailty can lead to disability and dependency [5,6], and become a burden to the individual, caregivers, and public health authorities. This process, which moves from robustness to frailty, disability, then dependency, is preventable, and could even improve in some aspects, if addressed in the early stages (i.e., in the prefrail state) [7,8,9,10].

Frailty, being a multifactorial condition, is related to several sociodemographic-, lifestyle-, and health-related factors. Associations were found between frailty risk and marital status [11], education [12], depression [13], polypharmacy [14], and nutritional status [2,15].

Gender discrepancies have also been reported regarding the risk of frailty. Although women tend to live longer, their health status is poorer than men and biological and socio-behavioral factors may contribute to a higher frailty predisposition [16,17]. In many studies, the prevalence and incidence of frailty in women was found to be greater than in men [18]. 

Among other risk factors for frailty, anthropometric, nutritional, and dietary risk factors are often mentioned [17]. Frailty is also closely related to body composition and nutritional status. It is well established that malnutrition and the risk of malnutrition, characterized by low mini-nutritional assessment (MNA) scores, increase the risk of frailty in older adults [19,20]. BMI was also associated with frailty in a U shape trend, with both low and high BMI being associated with higher incidence of frailty [21,22,23,24,25]. Furthermore, central obesity characterized by a high waist circumference (WC), has also been linked to frailty risk, and recent studies even reported that the association between BMI and frailty was in part mediated by waist to height ratio (WHTR) [26,27,28].

According to several authors, current recommended dietary allowances (RDA) for protein intake at 0.8 g/kg (BW) appears to be insufficient to prevent muscle loss with aging [29]. The protein intake that showed a better muscle preservation and was linked to lower frailty risk was proposed at protein intakes between 1 and 1.5 g/kg BW [29,30,31,32].

Apart from the nutritional status, dietary patterns were also linked with risk of frailty in older adults. Rashidi et al. showed that people adopting a healthy dietary pattern, characterized by a high consumption of fruits, vegetables, and whole grains, had 31% less chance of becoming frail [33]. Pooled results showed that a greater adherence to a Mediterranean diet, evaluated by the Mediterranean Diet Score (MDS), was associated with a significantly lower risk of frailty compared to poorer adherence [34,35]. Similar findings from the Hellenic Longitudinal Investigation on Aging and Diet (HELIAD) showed that each additional unit in the MDS was associated with a 5–7% decrease in the odds of frailty, depending on the tools used in the evaluation of frailty [36]. In Spain, a higher prevalence of frailty was observed in older adults adopting an unhealthy dietary pattern (DP) compared to individuals adopting a healthy dietary pattern [37]. In England, a longitudinal study showed that a dietary pattern characterized by a high fat intake and low fiber intake was associated with a higher risk of frailty in men. Following a prudent diet and having a higher adherence to a Mediterranean dietary pattern was also found to decrease the likelihood of frailty [38]. The Rotterdam cohort study showed that a higher adherence to national dietary guidelines was associated with lower risk of frailty over time, and a traditional dietary pattern characterized by a high consumption of legumes, eggs, and savory snacks was the only one protecting against frailty; health-conscious dietary patterns and high meat patterns failed to be associated with frailty [39]. Results from the 12-year follow-up, three-city study, showed that men following the dietary pattern characterized by a “pasta” pattern, and women adopting the “biscuits and snacking” pattern had a significantly higher risk of frailty compared with those following the “healthy” pattern (characterized by higher fish intake in men and higher fruits and vegetables intake in women) [40]. In the multicentric, 1 year NU-AGE interventional study, the administration of a Mediterranean diet for a 1year period, by modulating the microbiome ecosystem was linked with lower frailty incidence in older adults [41].

Several studies showed that prevalence of frailty and pre-frailty in low- to middle-income countries (LMIC) was higher than in high income countries (HIC) [18,42]. In Lebanon, one of the countries bordering the Mediterranean basin with an aging population, few studies have yet been interested in the frailty status among its elderly population. In 2016, the prevalence of frailty (including pre-frailty) was estimated to 66.8% in rural living older individuals, aged 65 years and more, with a higher prevalence of frailty among individuals considered undernourished or at risk of malnutrition according to MNA [19,43]. However, no data is available on the association between nutritional status, dietary patterns, and frailty in this population.

The aims of the present study were (i) to determine the prevalence of frailty and its covariates among older Lebanese adults, and (ii) to explore the association between dietary patterns and frailty among these individuals.

## 2. Materials and Methods

### 2.1. Study Design and Participants

We conducted a national, cross-sectional study in seven of the eight governorates of Lebanon. Recruitment of participants and data collection were carried out in collaboration with the Ministry of Social Affairs (MOSA) through 77 medico-social centers serving low to middle class income families, from October 2017 to October 2019. The sample was weighed according to the proportion of older adults over 60 years in each governorate and was randomly selected based on the sample previously drawn for the validation of the Arabic version of Mini-Mental State Examination (MMSE) [44,45].

**Figure 1:**
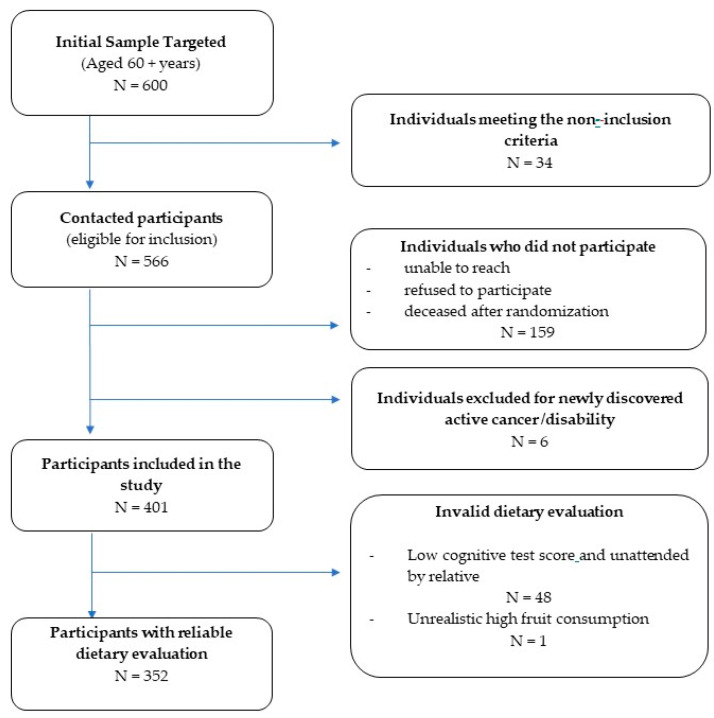
Study Flow chart.

As shown in Figure 1, a sample of 600 individuals was initially targeted. Individuals were included in the present study if they were aged 60 years and above, community dwelling, and attending the MOSA socio-medical centers for medical care and social assistance. Non-inclusion criteria included artificial feeding, total dependency, major hearing and visual impairment, active cancer disease, end stage kidney disease with hemodialysis, and advanced liver disease, as diagnosed by the center medical team. Once contact was initiated, 159 individuals were either unable to be reached, refused to participate, or were deceased after randomization, and six were excluded because of newly discovered cancer or major disability. Data were finally retained for 401 participants, of whom, 352 (88%) had a valid Food Frequency Questionnaire (FFQ).

Participants were contacted (via phone) by social workers or nurses working in the MOSA centers and were invited to attend the MOSA center. Information was collected by 10 dietitians living in the area surrounding the MOSA centers and who were familiar with the dietary habits of the local inhabitants. All investigators underwent several training sessions before starting the survey. To decrease investigator bias, all filled questionnaires were reviewed by the principal and field investigators before data entry. Each person was interviewed at the center near his/her home, and for participants unable to attend, the interview was performed by the research team at home. The interview with each participant or caretaker lasted around 30–45 min. Depending on literacy, the cognitive functions of each participant were assessed either through the Mini-Mental State Examination (MMSE) [44] or “Test des Neufs Images” (TNI) [46]. In case of significant cognitive decline, then the accompanying person was asked to fill the questionnaire on behalf of the participant.

The study was conducted in accordance with the Declaration of Helsinki, and the protocol of the study was approved by the Ethics Committee of Saint Joseph University of Beirut (USJ 2016-99). Written informed consent was signed by each participant, prior to completion of the interview.

### 2.2. Data Collected

#### 2.2.1. Frailty and FRAIL Scale

Frail status of the participants was assessed based on the five criteria of FRAIL scale including fatigue, resistance, ambulation, illness, and loss of weight [47]. Fatigue was measured by asking respondents how much time during the past 4 weeks they felt tired, with responses of “all of the time” or “most of the time” scoring 1 point. Resistance was assessed by asking participants if they had any difficulty walking up 10 steps alone without resting and without aids, and ambulation, by asking if they had any difficulty walking several hundred yards alone and without aids; “yes” responses were each scored as 1 point. Illness was scored 1 for respondents who reported 5 or more illnesses out of the 11 following illnesses: heart attack, congestive heart failure, angina, asthma, arthritis, stroke, kidney disease, hypertension, diabetes, cancer, and chronic lung disease. Weight loss was scored 1 for respondents with a self-reported weight decline of 5% or greater within the past 12 months. Frail scale scores range from 0–5 and represent frail (score = 3–5), pre-frail (score = 1–2), and robust (score = 0) health status [47]. For data analysis, the studied sample was further categorized to frail versus non-frail (robust or prefrail).

#### 2.2.2. Sociodemographic and Health-Related Data

Information regarding age and gender, living conditions, marital status, and economic situation were collected. Participants were asked to state whether they live alone or with any relative, spouse, family members, or friends. Educational level and literacy were evaluated and classified into two categories: >7 years and ≤7 years of education.

Number and type of diseases and health conditions were reported by each participant or its caregiver: cardiovascular diseases, diabetes, hypertension, heart failure and arrhythmia, renal disease, thyroid disorder, gastrointestinal diseases, anemia, osteoarthritis and arthritis, and osteoporosis. The presence of these diseases was ascertained based on: (a) self-reports linked to previous diagnoses, and/or (b) available medical records kept in the MOSA center, and/or (c) available medical prescriptions. The number of diseases was summed and categorized to multimorbidity defined as the simultaneous presence of two or more diseases in the same individual. Hearing or visual impairment, poor oral health, and sleep disorders were grouped as age-related conditions. Results were then dichotomized based on the presence of two or more of these age-related conditions. Current medications were recorded by caregiver, and polypharmacy was defined as the regular use of six or more prescription medications daily.

Since cognitive decline might influence reporting in the elderly population, cognitive impairment was evaluated depending on literacy, using population specific cut-offs based on age, educational level and sex, of MMSE for literate participants, and a score of TNI, Total Recall ≤ 9, for illiterate participants [44,46,48].

#### 2.2.3. Anthropometric and Functional Measurements

Data included height, weight, body mass index (BMI), waist circumference (WC), and waist to height ratio (WHR). BMI was categorized according to the Lipchitz classification (<22, 22–27, and >27 kg/m^2^) [49], Hand grip strength (HGS) was measured using the handgrip electronic dynamometer (Camry EH101). Handgrip strength was performed 3 times and the average of the three measurements was the reported result. We then used the BMI and gender specific cut-off values of HGS as classified by Fried phenotype, to classify as low or good HGS measurements [50].

Physical activity was evaluated using the Rapid Assessment of Physical Activity (RAPA) 2 scale. The nine-item questionnaire covered all ranges of activity, from sedentary to regular and vigorous physical activity in addition to strength training and flexibility adapted to the elderly [51]. Respondent’s score was initially categorized into five levels of physical activity. For data analysis, these levels were then classified into three categories: sedentary, regular active and optimal active. Responses to the strength training and flexibility items were scored separately [51].

#### 2.2.4. Nutritional and Dietary Data

Nutritional status was estimated using the Mini-Nutritional Assessment Short Form (MNA-SF) [52], classifying individuals into three levels: normal nutritional status (score above 12), at risk of malnutrition (scores 8–11), and malnutrition (scores below 8). Participants were then classified into two categories: the first category named “poor nutritional status” included participants that were considered malnourished and at risk of malnutrition by the MNA-SF, and the second category named “normal nutritional status” included participants who had a normal nutritional status according to MNA-SF.

Dietary assessment and food consumption was measured using a population based FFQ, reporting the consumption of a list of 90 food items (validation in progress of publication) and representing all food groups, consumed the previous year: bread and cereals, milk and dairy, vegetables and fruits, meat, poultry and fish, fats and oils, sweets and desserts, and non-alcoholic beverages. Consumption of these items was reported as daily, weekly, or monthly, as usual portion size. A manual illustrating the usual servings and portions of foods listed in the FFQ was developed for the study to help investigators and participants better estimate quantities consumed. The portions consumed were then translated into daily consumption in grams. Daily consumption was later analyzed by Nutrilog software (Nutrilog, version 3.20, France) to extract daily nutrient intake. To extract dietary patterns, the 90 foods listed in the FFQ were then grouped into 20 predefined categories based on similarities in nutrient composition and consumption characteristics (Supplemental Material: Table S1). These categories were then entered in the K-mean cluster analysis to determine the dietary patterns of our population.

For all the categories of food, except for sweets, reported portions used to estimate consumption were based on standard portions adopted in the dietary guidelines 2015–2020 [53]. Sweetened soft drinks were added to sugars and jams after sugar content estimation was made. As for sweets and desserts, usual serving size was adopted for cluster analysis.

### 2.3. Statistical Analysis

Statistical analysis was performed using the SPSS program version 21.0. Difference between genders and frailty status for sociodemographic, nutritional, dietary, health-related, and anthropometric data, were compared using the non-parametric tests, Mann–Whitney and Kruskal–Wallis for numeric variables that are not normally distributed, and chi-square test for categorical variables. K-means clustering was used to regroup participants with similar dietary patterns. Differences in food intake between dietary patterns was calculated by Kruskal–Wallis test. Cut-offs for age, polypharmacy, multimorbidity, age-related conditions, and waist to height ratio (WHTR) were determined through classification tree and used for subsequent multivariate analysis. In the multivariate analyses, we proceeded to several models of binary logistic regression using dichotomized frailty variable as the dependent variable. Odds-ratios with 95% confidence intervals were calculated. The main explanatory variables reaching significance level identified in each model were added to the following model. Age category, marital status, education level, and living conditions were entered first (model 1). From this model, we retained covariates significantly associated with frailty and then added anthropometric variables including MNA-SF, BMI, and waist to height ratio categories (model 2). From model 2, we retained all covariates significantly associated with frailty and then added health-related covariates (multimorbidity, polypharmacy, and age-related conditions (model 3). From model 3, we retained all covariates significantly associated with frailty and finally added food dietary patterns (model 4). Each Model was rerun with the food patterns groups in order to adjust for the variables identified respectively in each model. The multivariate analysis was rerun for each gender. The level of significance was fixed at a *p* = 0.05 for all analyses.

## 3. Results

### 3.1. Sample Characteristics

The present analysis included 352 participants, with 50% being women. Details of the sample characteristics are shown in Table 1. 

The sample accounted for 15.1% frail individuals, with more women being classified as frail compared to men: 35 (19.9%) and 18 (10.2%), respectively. In addition, women had more age-related conditions compared to men and more women than men had a low educational level.

Median BMI for the total sample was 28.2 kg/m^2^, with 60.5% of the sample having a BMI > 27 kg/m^2^. Women had a significantly higher median BMI 29.7 (25.9–35.1) kg/m^2^ compared to men 27.5 (24.3–31.2) kg/m^2^. Median WHTR was also significantly higher among women. With 63.9% of the sample having a normal nutritional status as evaluated by MNA-SF, no difference was found between nutritional statuses among gender.

Median energy intake of the participants was 1824 Kcal/d. Energy, carbohydrates, and protein intakes were lower in women compared to men. Median protein intake per kg body weight (g/kg BW) was also lower in women (0.85 g/kg BW) compared to men (0.94 g/kg BW). Caloric intake per kg body weight (Kcal/kg BW) was nonetheless not different between the two sexes.

### 3.2. Dietary Patterns

As shown in Table 2, three dietary patterns were identified in the total sample. The first, named Westernized-type dietary pattern (WDP), followed by 11.9% of the participants (29 men and 13 women), was characterized by the highest caloric intake, consumption of refined flour products, sugar and sweets, dairy products, as well as processed and saturated fats and the lowest olive, seeds, and oleaginous fruits and whole cereal products intake. The second pattern, named high intake/Mediterranean-type dietary pattern (HI-MEDDP), adopted by 23% of participants (21 men and 60 women), was characterized by a relatively high caloric intake, a higher consumption of vegetables, fruits, legumes than the other 2 DPs, and the highest consumption of foods rich in monounsaturated fats. Median consumption of olive, seeds, and oleaginous fruits in the HI-MEDDP group was above nine teaspoons of oil equivalent per day. This pattern also had the lowest consumption of refined flour products, and the highest consumption of whole cereal products. Finally, the third pattern, named moderate intake/Mediterranean-type dietary pattern (MOD-MEDDP), represented the highest proportion of our sample (65.1% of the sample, with 126 men and 106 women), and was characterized by a diversified and balanced DP. Consumption of most foods in this pattern was either intermediate or lower compared to the other two patterns, with the lowest consumption of sweets and sugar among the three patterns.

Gender-specific food consumption characteristics of the 3 DP, showed that women following the WDP consumed more sugar portions (median consumption of 13 teaspoons of sugars and jams) than men (equivalent to almost seven teaspoons of added sugar) in this same pattern, although median energy intake was on average lower in women compared to men. Men and women adopting the HI-MEDDP had the highest median consumption of olive, seeds, and oleaginous fruits (12 and 9 teaspoons equivalent of fat, respectively) (data in Supplementary Table S2).

### 3.3. Frailty

#### 3.3.1. Frailty Association with Sociodemographic and Health-Related Factors

Sociodemographic, health, and nutritional characteristics of the study sample according to the frailty status are represented in Table 3. Factors associated with frailty included higher age, female gender, and lower educational level (<7 years at school); the latter was not associated to frailty in men.

Taking more than five medications, having more than one disease, more than one age-related condition, and having a higher WHTR was associated with frailty in the total sample and in women. In men, only multimorbidity was found to be associated with frailty, whereas no association with health and anthropometric parameters was observed.

Regarding nutritional status evaluated by MNA-SF, we identified a significant association with frailty, with a higher prevalence of poor nutritional status (malnutrition and risk of malnutrition) among frail individuals in both genders.

#### 3.3.2. Frailty and Dietary Patterns

Table 4 displays the dietary patterns and food intake associated with frailty. 

Individuals adopting the MOD-MEDDP accounted for 67.2% of the non-frail group compared to 21.7% and 11%, for the HI-MEDDP and WDP, respectively. Nonetheless, in the univariate analysis, dietary patterns seemed to be associated with frailty status only in women. Women adopting the WDP accounted for 5% of the non-frail group compared to 17.1% of the frail group, and women adopting the MOD-MEDDP accounted for 63.1% of the non-frail group compared to 40% of the frail group.

Concerning nutrients, median caloric, carbohydrates, and protein intakes were higher in the non-frail compared to frail group, in the total population, and in men. Median g of protein/kg BW was found to be significantly higher in the non-frail group, only in the overall population.

The multivariate logistic regression analysis performed in the total sample (Table 5), included 327 individuals with complete set of data each, and fulfilling all criteria of inclusion. Within the total sample, in the first model, independent factors associated with frailty, included age above 75 years, female gender, and low level of education. When anthropometric and nutritional status parameters were added in the second model, factors associated with frailty were age above 75 years, WHTR > 0.718 and poor nutritional status compared to normal nutritional status. In the third model, when health-related parameters were added to the analysis, factors associated with frailty included age, WHTR > 0.718, poor nutritional status, polypharmacy, multimorbidity, and age-related conditions. In the final model, by adding dietary patterns, independent factors comprised age above 75 years, WHTR > 0.718, poor nutritional status, polypharmacy, age-related conditions, and WDP.

The multivariate association between dietary patterns and frailty is described in Table 6. In the overall sample, no association was observed between dietary patterns and frailty prevalence. After adjusting for main confounders, women adopting the WDP, compared to those adopting the MOD-MEDDP, exhibited a higher prevalence of frailty, in the first and second models, as well as in the fully adjusted model (odds ratio ((OR) 11.54, 95% confidence interval (CI) (2.02–65.85)). In men, similar results were observed: adopting a WDP was associated with a higher prevalence of frailty only in the fully adjusted model, ((OR) 6.63, 95% (CI) (1.82–24.21)), when compared to the MOD-MEDDP. Finally, the HI-MEDDP was not significantly associated with frailty prevalence, in the overall sample, nor in men compared with following a MOD-MEDDP.

## 4. Discussion

Our study aimed at describing the prevalence of frailty and its associated factors, including dietary patterns, in a national sample of community dwelling older Lebanese individuals. In this rural and urban low socioeconomic sample, the estimated prevalence of frailty was 15% on average, with 10% and 20% in men and women, respectively. In men, poor nutritional status and being older than 75 years were found to be associated with frailty. In women, in addition to these two factors, taking more than five drugs daily, having at least one age-related condition, having a WHTR > 0.718 and following a Westernized-type DP were found to be independently associated with frailty. In women, the HI-MEDDP showed a significant association with frailty prevalence after adjusting for age, educational level, nutritional status, and WHTR. However, this failed to be significant after further adjusting for polypharmacy and age-related conditions.

Our frailty prevalence can be compared to findings of a meta-analysis conducted in 2018, where pooled prevalence of frailty was 17.4% [42]. A Lebanese national study involving 1200 individuals reported a higher prevalence of frailty, at 36.4%, in an exclusively rural elderly population. In this previous study, 73% of the sample had a monthly income below the minimum wage, whereas in our studied sample, 57.8% had low or insufficient income [19]. The difference with our results can be explained by the variability in study design and tools used for the evaluation of frailty, as well as the heterogeneity between rural and urban settings. Rural areas, being usually poorer and more affected by urban migration of young adults, might witness a higher proportion of frail individuals. [45,54,55,56].

As shown in previous research, women are more often frail than men [54]. Our results also showed that a low educational level (less than 7 years) was associated with prevalence of frailty, particularly in women. As described in the Longitudinal Aging Study in Amsterdam, the impact of low educational level increased the odds of frailty almost three-fold and this association persisted throughout the 13 years of follow-up [57]. Health related parameters were also found to be associated with frailty, particularly in women; they were frailer if they were taking more than five drugs every day, suffered from two or more diseases, and had at least one age-related condition. These factors have been often linked to frailty in several settings [14,58,59,60,61,62].

WHTR was associated with frailty, in the multivariate analysis, suggesting a possible role of higher abdominal adiposity. Although low BMI is known to be associated with frailty, obesity is also considered as risk a factor of frailty [24]. A meta-analysis showed that overweight individuals (BMI between 25–30 kg/m^2^) exhibited an increased risk of frailty by 20%, whereas obese (BMI ≥ 30), have an increased frailty risk of 90% [21]. In the longitudinal Doetinchem Study, a BMI < 23 kg/m^2^ and ≥30 kg/m^2^ was associated with higher incidence of frailty [25]. Similar results were found in the Japanese cohort with a lowest incidence of frailty at a BMI between 21.4 and 25.7 kg/m^2^ [22]. As shown by Kim et al., the risk of frailty is higher in obese women, which is mediated by WHTR, but not in obese men [28]. In Spain, two cohort studies showed a parallel change of abdominal obesity and BMI to be associated with an increasing risk of frailty [63].

The level of malnutrition was identified as an independent risk factor associated with frailty, although our sample had a high proportion of obese. Our study showed that malnutrition was associated with a substantially increased prevalence of frailty in the total sample and in both gender groups. The relation between malnutrition and frailty was already clearly established in previous studies, and overweight and obesity often co-exist with frailty [21,22,27,28,63,64,65]. 

Among the three dietary patterns identified in our study, the WDP was associated with a higher prevalence of frailty, in both men and women, independently of major confounding factors. This pattern was characterized by a high median sugar intake particularly in in women, and the highest consumption among the three patterns in refined flour products. Several studies reported a link between sugar consumption and the risk of frailty. In the 5-year cohort Seniors-ENRICA study, a high consumption of added sugar, ≥ 36 g/day, compared to <15 g/day, was found to increase the odds of frailty by almost two-fold [66]. Furthermore, in the Nurses’ Health Study, after adjustment for confounding factors, consumption of ≥2 servings of sugar-sweetened beverages per day compared to no consumption, increased the risk of frailty by almost 30% over a period of 22 years [67].

Healthy moderate DP and Mediterranean DP were often reported to be inversely associated with frailty [34,36,68,69]. Frailty risk was also found to be inversely associated with consumption of fruits and vegetables [70,71,72]. Our analysis showed that the Mediterranean dietary pattern with moderate intakes, represented by the MOD-MEDDP in our study, had the lowest prevalence of frailty. On the other hand, when HI-MEDDP group was compared to MOD-MEDDP in women, the predominance of obesity in this group, with a concomitant high fat intake (the highest among the 3 DPs) and a median protein intake of 0.9 g/kg BW, may have contributed to frailty in this subcategory. This could suggest that these conditions, regardless of the quality of fat and diet, could outweigh the beneficial effect of a Mediterranean diet on the prevalence of frailty. Previous reports suggested that protein intake between 1 and 1.5 g/kg BW were necessary for the prevention of frailty [29,30,31,32].

In summary, our study was the first to explore the association between frailty and specific dietary patterns extracted in a posteriori method, in adults over 60 years of age in Lebanon, using specifically validated questionnaires. Despite the difficulties in addressing this specific age group, we succeeded in shedding light on some findings specific to our Mediterranean older adult population. Age, age-related conditions, polypharmacy, and malnutrition, remain the main associated factors related to frailty in low socioeconomic settings. We also showed that malnutrition and abdominal obesity co-exist as risk factors for frailty. Most importantly, we demonstrated that a Westernized-type pattern with high sugar consumption, and to a lesser extent, a Mediterranean high caloric intake pattern, were also linked to frailty, and that a more moderate Mediterranean-like pattern was protective, especially in women. More efforts should target actions that improve modifiable factors to prevent or reverse frailty, such as eating patterns and diets that improve WHTR.

We note, however, some limitations concerning our results in relation to the low number of frail participants adopting the WDP; this consequently implies taking the present findings with caution.

Larger prospective studies are required to further investigate the impact of dietary patterns on risk of frailty with a special emphasis on WHTR.

In conclusion, WDP had the strongest association with frailty in this sample. MOD-MEDDP, in comparison to HI-MEDDP and WDP, was associated with the least prevalence of frailty. In this Mediterranean sample, a diet far from the traditional one appears as a key deleterious determinant of frailty.

## Figures and Tables

**Table 1:** Sociodemographic and clinical characteristics of the total sample, and stratified by gender (*N* = 352).

		Total (*N* = 352)	Men (*N* = 176)	Women (*N* = 176)	*p* Value
**Sociodemographic Status**					
**Age (years)**		73 (67–79)	73 (67.3–80)	73 (67–78)	0.288
**Marital status**	Married	232 (65.9)	143 (81.3)	89 (50.6)	<0.001
	Divorced	10 (2.8)	4 (2.3)	6 (3.4)	
	Single	18 (5.1)	6 (3.4)	12 (6.8)	
	Widowed	92 (26.1)	23 (13.1)	69 (39.2)	
**Living conditions**	Living Alone	53 (15.1)	16 (9.1)	37 (21)	<0.001
	Living with partner	202 (57.4)	131 (74.4)	71 (30.3)	
	Living with others	97 (27.6)	29 (16.5)	68 (38.6)	
**Education**	Low level	149 (42.3)	65 (36.9)	84 (47.7)	0.04
**Economic status**	Insufficient income	198 (56.3)	98 (55.7)	100 (56.8)	0.159
**Health and Functional Status**					
**Frailty status**	Frail	53 (15.1)	18 (10.2)	35 (19.9)	0.01
	Not frail	299 (84.9)	158 (89.8)	141 (80.1)	
**Cognitive Status**	Low cognitive test	116 (33)	57 (32.4)	59 (33.5)	0.821
**Polypharmacy**	≥6 drugs	254 (73.8)	133(77.8)	121 (69.9)	0.098
**Multi-morbidity**	≥2 chronic illnesses	270 (76.9)	127 (72.6)	143 (81.3)	0.054
**Age-related conditions**	≥2 conditions	189 (53.7)	84 (47.7)	105 (59.7)	0.025
**Physical Activity**	Sedentary	275 (78.1)	146 (83)	129 (73.3)	0.301
	Regular Active	74 (21)	30 (17)	44 (25)	
	Optimal active	3 (0.9)	0 (0)	3 (1.7)	
**Strength**	HGS (kg)	23.1 (17.8–31.2)	31 (24.5–35.7)	18.3 (13.6–22.3)	<0.001
**Nutritional Status and Nutrient Intake**					
**BMI**	BMI(kg/m^2^)	28.2 (25–32.8)	27.5 (24.3–31.2)	29.7 (25.9–35.1)	<0.001
	BMI < 22	109 (31)	64 (36.4)	45 (25.6)	0.042
	BMI (22–27)	30 (8.5)	17 (9.7)	13 (7.4)	
	BMI > 27	213 (60.5)	95 (54)	118 (67)	
**WTHR**		0.64 (0.59–0.70)	0.62 (0.57–0.67)	0.67 (0.61–0.75)	<0.001
**Nutritional status**	Normal	225 (63.9)	114 (64.8)	111 (63.1)	0.74
**(MNA-SF score)**	Poor	127 (36.1)	62 (35.2)	65 (36.9)	
**Energy and macronutrients intake**	Energy (Kcal/d)	1824 (1509–2299)	2031 (1638–2447)	1726 (1408–2094)	<0.001
	Calories (Kcal/kg BW)	25.3 (20.1–31.8)	26.1 (20.1–31.8)	24.5 (19.5–31.9)	0.214
	Carbohydrates(g/d)	201 (161.3–258)	226 (178.5–293)	185.5 (145.3–218.5)	<0.001
	Proteins (g/d)	66.7 (50.1–84.1)	72.3 (58.9–92.3)	59.6 (46.1–73.2)	<0.001
	Proteins (g/kg BW)	0.91 (0.68–1.15)	0.94 (0.73–1.17)	0.85 (0.65–1.13)	0.022
	Fats (g/d)	89.2 (72.5–111)	90.4 (74.1–112.8)	86.3 (70.6–109.8)	0.076

Abbreviations: BMI: body mass index; WC: waist circumference; WHTR: waist to height ratio; HSG: handgrip strength; Kcal/d: Calories per day; Calories/kg BW: Calories per kg body weight; Protein/kg BW: Proteins per kg body weight. Numeric variables are represented as median (interquartile range). Categorical variables are represented as *N* (percentage).

**Table 2:** Consumption of predefined food categories according to dietary patterns for the overall sample. *N* = 352.

	WDP	HI-MEDDP	MOD-MEDDP	*p* Value
	*N* = 42 (11.9%)	*N* = 81 (23%)	*N* = 229 (65.1%)	
Kilocalories/day	2261 (1953–2845)	2028 (1652–2473)	1743 (1453–2123)	<0.001
Refined flour products	5.09 (3.38–7.84)	2.25 (0.7–5.09)	3.32 (1.15–5.11)	<0.001
Whole breads and cereals (including burghul)	0.35 (0.11–1.46)	1.49 (0.36–2.82)	0.76 (0.18–2.33)	0.008
Potato	0.4 (0.14–0.78)	0.27 (0.1–0.51)	0.25 (0.1–0.43)	0.024
Vegetables	2.9 (2.1–3.84)	3.74 (2.59–5.23)	2.93 (2.09–4.52)	0.002
Fruits	2.1 (1.09–2.78)	2.45 (1.52–3.4)	1.89 (1.25–2.86)	0.033
Legumes	0.3 (0.18–0.57)	0.57 (0.24–0.86)	0.29 (0.13–0.57)	0.006
Meat and poultry	2.26 (1.49–3.6)	2.02 (1.5–2.94)	1.93 (1.2–3.03)	0.137
Eggs	0.57 (0.25–0.86)	0.29 (0.18–0.57)	0.29 (0.1–0.57)	0.002
Fish and shellfish	0.42 (0.08–0.78)	0.43 (0.17–0.83)	0.4 (0.13–0.73)	0.568
Milk and dairy products	1.89 (1.05–2.61)	1.71 (0.99–2.43)	1.39 (0.85–2.05)	0.002
Vegetable oils	3 (0.94–4)	3 (1–3.62)	3 (2–3.08)	0.704
Olive, seeds and oleaginous fruits	4.03 (3–6.04)	9.87 (8.02–12.67)	4.16 (3.07–6.18)	<0.001
Processed and saturated fats	0.02 (0–1)	0 (0–0.17)	0 (0–0.18)	0.024
Low fat sweets	0.49 (0.07–2.11)	0.43 (0.08–1.33)	0.14 (0–0.36)	<0.001
High fat sweets	0.19 (0.07–0.49)	0.14 (0–0.43)	0.07 (0–0.26)	<0.001
Sugars and jams	8.5 (6.05–12.36)	3 (0.96–5.37)	1 (0.15–2.13)	<0.001

Abbreviations: WDP: Westernized dietary pattern; HI-MEDDP: high-intake Mediterranean dietary pattern; MOD-Med: moderate-intake Mediterranean dietary pattern. Caloric intake and intake of food groups per day are represented as Median (interquartile range). Values represent the cluster centers of each food group in the three identified food patterns, expressed in portions/day.

**Table 3:** Sociodemographic and health-related factors associated with frailty in total sample and stratified by gender.

		Total			Men			Women		
		*N* = 352			176 (50)			176 (50)		
		Non-Frail	Frail	*p Value*	Non-Frail	Frail	*p Value*	Non-Frail	Frail	*p Value*
N (%)		299 (84.9)	53 (15.1)		158 (89.8)	18 (10.2)		141 (80.1)	35 (19.9)	
**Sociodemographic Parameters**										
Age (years)		72 (60–93)	78 (60–91)	<0.001	72 (60–93)	82 (60–88)	0.003	72 (60–90)	76 (65–91)	0.001
Education	Elementary and lower	116 (38.8)	33 (62.3)	0.001	57 (36.1)	8 (44.4)	0.486	59 (41.8)	25 (71.4)	0.002
**Health-Related Parameters**										
Polypharmacy		61 (21)	29 (54.7)	<0.001	32 (20.9)	6 (33.3)	0.231	29 (21)	23 (65.7)	<0.001
Multi-morbidity		218 (73.2)	52 (98.1)	<0.001	110 (70.1)	17 (94.4)	0.028	108 (76.6)	35 (100)	<0.001
Age-related conditions		148 (49.5)	41 (77.4)	<0.01	74 (46.8)	10 (55.6)	0.483	74 (52.5)	31 (88.6)	<0.001
**Anthropometric Parameters**										
BMI (kg/m^2^)		28.1 (16.5–49.4)	29.8 (18.5–46.2)	0.096	27.5 (16.5–40)	26.1 (18.5–34)	0.173	28.9 (18.2–49.4)	32.4 (19.6–46.1)	0.018
WHTR		0.64 (0.42–0.99)	0.69 (0.49–1)	<0.001	0.62 (0.42–0.41)	0.6 (0.52–0.69)	0.705	0.65 (0.5–0.99)	0.75 (0.49–1)	<0.001
**Nutritional Status, Strength, and Activity**										
Low HGS		127 (45)	44 (86.3)	<0.001	64 (43)	13 (81.2)	0.004	63 (47.4)	31 (88.6)	<0.001
Physical activity	Sedentary	224 (74.9)	51 (96.2)	<0.001	128 (81)	18 (100)	0.042	96 (68.1)	33 (94.3)	<0.001
	Regular	72 (24.1)	2 (3.8)		30 (19)	0 (0)		42 (29.8)	2 (5.7)	
	Optimal	3 (1)	0 (0.0)		0 (0)	0 (0)		3 (2.1)	0 (0)	
Poor nutritional status		96 (32.1)	31 (58.5)	<0.001	51 (32.3)	10 (55.6)	<0.001	44 (31.2)	21 (60)	<0.001

Abbreviations: BMI: body mass index; WC: waist circumference; WHTR: waist to height ratio; HSG: handgrip strength; MNA-SF: Mini-Nutritional Assessment-Short Form. Numeric variables are represented as median (interquartile range). Categorical variables are represented as *N* (%).

**Table 4:** Dietary patterns, food, and nutrient intake associated with frailty in the total sample and stratified by gender.

	Total (*N* = 352)			Men (*N* = 176)			Women (*N* = 176)		
	Non-Frail 299 (85)	Frail 53 (15)	*p*	Non-Frail 158 (90)	Frail 18 (10)	*p*	Non-Frail 141 (80)	Frail 35 (20)	*p*
**Food Patterns**									
**WDP**	33 (11)	9 (17)	0.125	26 (16.5)	3 (16.7)	0.673	7 (5)	6 (17.1)	0.01
**HI-MEDDP**	65 (21.7)	16 (30.2)		20 (12.7)	1 (5.6)		45 (31.9)	15 (42.9)	
**MOD-MEDDP**	201 (67.2)	28 (52.8)		112 (70.9)	14 (77.8)		89 (63.1)	14 (40)	
**Food Intake**									
**Sugars and jams**	1.8 (0.29–4)	2 (0.36–5.5)	0.21	1.64 (0.32–4)	2.14 (0.25–4)	0.794	1.93 (0.3–3.75)	1.85 (0.43–6.7)	0.204
**Low fat sweets**	0.17 (0.01–0.59)	0.3 (0.08–1.08)	0.037	0.13 (0–0.47)	0.15 (0.04–0.5)	0.53	0.24 (0.04–0.8)	0.37 (0.14–1.3)	0.097
**Fruits and Vegetables**	5.6 (4.1–7.4)	4.8 (3.1–6.6)	0.022	5.8 (4.08–7.35)	4.7 (3.8–6.9)	0.196	5.6 (4.2–7.5)	5.2 (3.1–6.4)	0.07
**Vegetable oils**	3 (1.5–3.1)	3 (2.5–4)	0.027	3 (1.5–3.16)	3 (1–3)	0.96	3 (1.5–3.08)	3 (3–4)	0.009
**Olive, seeds and oleaginous fruits**	5.04 (3.3–7.7)	6 (3.6–8.3)	0.513	5.2 (3.3–7.5)	5.1 (3.96–6.7)	0.786	4.8 (3.4–7.82)	6.4 (3.4–9.1)	0.305
**Energy and Macronutrients**									
**Caloric intake** **(Cal/d)**	1851 (1543–2349)	1763 (1398–2075)	0.039	2077.5 (1667–2487)	1734 (1422–1900)	0.025	1709 (1416–2082)	1763 (1388–2120)	0.859
**Kilocalories/kg BW**	25.63 (20.1–32.1)	23.1 (19.4–30.3)	0.095	26.5 (21.03–32.1)	22.4 (18.2–29.9)	0.107	24.93 (19.84–31.9)	23.7 (19.4–31.7)	0.457
**Carbohydrates (g)**	204 (166–263)	184 (143–217)	0.009	237.5 (181–295)	198.5 (152–233)	0.038	188 (149–219)	167 (129–217)	0.356
**Proteins (g)**	67.8 (51.3–84.8)	57.4 (44.9–70.5)	0.006	74.05 (60.4–94.1)	62 (51.8–84.6)	0.033	61.1 (46.3–77.4)	54.4 (44.1–70.5)	0.295
**Protein/kg BW**	0.93 (0.69–1.17)	0.81 (0.66–0.97)	0.016	0.96 (0.74–1.18)	0.81 (0.73–1.07)	0.133	0.87 (0.65–1.16)	0.81 (0.63–1)	0.161
**FAT (g)**	89.5 (74.1–111)	84.9 (66.2–113)	0.328	91.65 (75.8–115)	81.25 (63.8–107)	0.058	85.8 (70.7–107)	90.9 (70–119)	0.638

Abbreviations: WDP: Westernized dietary pattern. HI-MEDDP: high-intake Mediterranean dietary pattern. MOD-Med: moderate-intake Mediterranean dietary pattern. Kcal/d: calories per day. Calories/kg BW: calories per kg body weight. Protein/kg BW: proteins per kg body weight. Numeric variables are represented as median (interquartile range). Categorical variables are represented as *N* (%).

**Table 5:** Binary logistic regression models of frail vs. non-frail for the total sample.

Associated Factors	Model 1	Model 2	Model 3	Model 4
	**Sociodemographic** **Parameters**			
**Age > 75 years**	**3.667 (1.93–6.99)**	**3.55 (1.75–7.21)**	**2.91(1.42–5.94)**	**2.83(1.42–5.63)**
**Female gender**	**2.66 (1.33–5.29)**	2.11 (0.99–4.49)		
**Low Education level (<7 years of education)**	**2.19 (1.17–4.11)**	1.96 (0.98–3.93)	1.93 (0.94–3.97)	
**Living conditions (compared with living alone)**				
**with partner**	0.64 (0.26–1.59)			
**with others**	1.14 (0.35–3.7)			
**Marital status (married vs. other status)**	1.95 (0.78–4.9)			
		**Nutritional and** **Anthropometric** **Parameters**		
**WHTR>0.718**		**2.94 (1.27–6.76)**	**3.27 (1.56–6.83)**	**3.78 (1.71–8.33)**
**Poor nutritional status (malnutrition and at risk vs normal)**		**14.26 (4.64–43.81)**	**10.79 (3.29–35.35)**	**9.67 (3.1–30.18)**
**BMI (compared with BMI < 22 kg/m^2^)**				
**BMI (22–27)**		0.72 (0.16–3.25)		
**BMI (>27)**		1.24 (0.52–2.98)		
			**Health-Related Parameters**	
**Polypharmacy**			**2.74 (1.34–5.6)**	**4.42 (2.21–8.86)**
**Age related conditions**			**2.28 (1.03–5.03)**	**2.47 (1.17–5.24)**
**Multimorbidity**			7.18 (0.92–56.03)	
				**Dietary Patterns**
**MOD-MEDDP pattern**				1
**WDP pattern**				**2.97 (1.12–7.89)**
**HI-MEDDP pattern**				2.27 (0.98–5.25)

Note: Bold values are statistically significant, *p* < 0.05.

**Table 6:** Multivariate associations between dietary patterns and frailty prevalence. Beirut 2021, *N* = 327.

	Dietary Patterns	Frail Individuals N (%)	Model 1		Model 2		Model 3	
		53 (15)	OR (95% CI)	*p*	OR (95% CI)	*p*	OR (95% CI)	*p*
**Total**				0.176		0.074		0.083
	**MOD-MEDDP**	28 (52.8)	1		1		1	
	**WDP**	9 (17)	2.25(0.91–5.53)	0.078	2.44 (0.93–6.43)	0.07	2.68 (0.98–7.29)	0.054
	**HI-MEDDP**	16 (30.2)	1.45 (0.7–2.98)	0.316	2.08 (0.96–4.54)	0.065	1.96 (0.86–4.45)	0.109
**Men**				0.482		0.735		0.011
	**MOD-MEDDP**	14 (77.8)	1		1		1	
	**WDP**	3 (16.7)	1.14 (0.29–4.53)	0.85	0.89 (0.18–4.39)	0.885	6.63 (1.82–24.21)	0.004
	**HI-MEDDP**	1 (5.6)	0.28 (0.03–2.35)	0.243	0.43 (0.05–3.62)	0.434	2.23 (0.93–5.32)	0.071
**Women**				0.027		0.013		0.013
	**MOD-MEDDP**	14 (40)	1		1		1	
	**WDP**	6 (17.1)	4.57 (1.25–16.76)	0.022	6.76 (1.56–29.22)	0.01	11.54 (2.02–65.85)	0.006
	**HI-MEDDP**	15 (42.9)	2.44 (1.02–5.79)	0.044	3.06 (1.15–8.15)	0.025	3.06 (0.97–9.62)	0.056

Model 1: model adjusted for age, gender, educational level. Model 2: model 1 further adjusted for nutritional status, and WHTR. Model 3: model 2 further adjusted for polypharmacy and age-related conditions.

## Supplementary Materials

The following are available online at https://www.mdpi.com/article/10.3390/nu13072188/s1, Table S1: food groups and food items included in the cluster analysis, Table S2: consumption of predefined food categories according to dietary patterns for men and women separately, Table S3: nutritional characteristics of the dietary patterns.

## References

[1] Fried L.P., Tangen C.M., Walston J., Newman A.B., Hirsch C., Gottdiener J., Seeman T., Tracy R., Kop W.J., Burke G. (2001). Frailty in Older Adults: Evidence for a Phenotype. J. Gerontol. A Biol. Sci. Med. Sci..

[2] Verlaan S., Ligthart-Melis G.C., Wijers S.L.J., Cederholm T., Maier A.B., de van der Schueren M.A.E. (2017). High Prevalence of Physical Frailty Among Community-Dwelling Malnourished Older Adults—A Systematic Review and Meta-Analysis. J. Am. Med. Dir. Assoc..

[3] Crow R.S., Lohman M.C., Pidgeon D., Bruce M.L., Bartels S.J., Batsis J.A. (2018). Frailty Versus Stopping Elderly Accidents, Deaths and Injuries Initiative Fall Risk Score: Ability to Predict Future Falls. J. Am. Geriatr. Soc..

[4] Marques A., Queirós C., Hertz K., Santy-Tomlinson J. (2018). Frailty, Sarcopenia and Falls. Fragility Fracture Nursing.

[5] Morley J.E. (2017). The New Geriatric Giants. Clin. Geriatr. Med..

[6] Kojima G. (2017). Frailty as a Predictor of Disabilities among Community-Dwelling Older People: A Systematic Review and Meta-Analysis. Disabil. Rehabil..

[7] Nwagwu V.C., Cigolle C., Suh T. (2020). Reducing Frailty to Promote Healthy Aging. Clin. Geriatr. Med..

[8] Lorenzo-López L., López-López R., Maseda A., Buján A., Rodríguez-Villamil J.L., Millán-Calenti J.C. (2019). Changes in Frailty Status in a Community-Dwelling Cohort of Older Adults: The VERISAÚDE Study. Maturitas.

[9] Zamudio-Rodríguez A., Letenneur L., Féart C., Avila-Funes J.A., Amieva H., Pérès K. (2020). The Disability Process: Is There a Place for Frailty?. Age Ageing.

[10] Fried L.P., Cohen A.A., Xue Q.-L., Walston J., Bandeen-Roche K., Varadhan R. (2021). The Physical Frailty Syndrome as a Transition from Homeostatic Symphony to Cacophony. Nat. Aging.

[11] Kojima G., Walters K., Iliffe S., Taniguchi Y., Tamiya N. (2020). Kojima Gotaro Marital Status and Risk of Physical Frailty: A Systematic Review and Meta-Analysis. J. Am. Med. Dir. Assoc..

[12] Kingston A., Davies K., Collerton J., Robinson L., Duncan R., Kirkwood T.B.L., Jagger C. (2015). The Enduring Effect of Education-Socioeconomic Differences in Disability Trajectories from Age 85 Years in the Newcastle 85+ Study. Arch. Gerontol. Geriatr..

[13] Soysal P., Veronese N., Thompson T., Kahl K.G., Fernandes B.S., Prina A.M., Solmi M., Schofield P., Koyanagi A., Tseng P.T. (2017). Pinar Soysal Relationship between Depression and Frailty in Older Adults: A Systematic Review and Meta-Analysis. Ageing Res. Rev..

[14] Gutiérrez-Valencia M., Izquierdo M., Cesari M., Casas-Herrero Á., Inzitari M., Martínez-Velilla N. (2018). The Relationship between Frailty and Polypharmacy in Older People: A Systematic Review: Frailty and Polypharmacy: A Systematic Review. Br. J. Clin. Pharmacol..

[15] Lorenzo-López L., Maseda A., de Labra C., Regueiro-Folgueira L., Rodríguez-Villamil J.L., Millán-Calenti J.C. (2017). Nutritional Determinants of Frailty in Older Adults: A Systematic Review. BMC Geriatr..

[16] Hubbard R.E. (2015). Sex Differences in Frailty. Frailty Aging.

[17] Zhang Q., Guo H., Gu H., Zhao X. (2018). Gender-Associated Factors for Frailty and Their Impact on Hospitalization and Mortality among Community-Dwelling Older Adults: A Cross-Sectional Population-Based Study. PeerJ.

[18] Ofori-Asenso R., Chin K.L., Mazidi M., Zomer E., Ilomaki J., Zullo A.R., Gasevic D., Ademi Z., Korhonen M.J., LoGiudice D. (2019). Global Incidence of Frailty and Prefrailty Among Community-Dwelling Older Adults. JAMA Netw. Open.

[19] Boulos C., Salameh P., Barberger-Gateau P. (2016). Malnutrition and Frailty in Community Dwelling Older Adults Living in a Rural Setting. Clin. Nutr..

[20] Kizilarslanoglu M.C., Sumer F., Kuyumcu M.E. (2016). Malnutrition Increases Frailty among Older Adults: How?. Clin. Nutr..

[21] Amiri S., Behnezhad S., Hasani J. (2020). Amiri Sohrab Body Mass Index and Risk of Frailty in Older Adults: A Systematic Review and Meta-Analysis. Obes. Med..

[22] Watanabe D., Yoshida T., Watanabe Y., Yamada Y., Kimura M. (2020). A U-Shaped Relationship between the Prevalence of Frailty and Body Mass Index in Community-Dwelling Japanese Older Adults: The Kyoto–Kameoka Study. J. Clin. Med..

[23] Crow R.S., Petersen C.L., Cook S.B., Stevens C.J., Titus A.J., Mackenzie T.A., Batsis J.A. (2020). Weight Change in Older Adults and Risk of Frailty. J. Frailty Aging..

[24] Tabue-Teguo M., Perès K., Simo N., Le Goff M., Perez Zepeda M.U., Féart C., Dartigues J.-F., Amieva H., Cesari M. (2020). Gait Speed and Body Mass Index: Results from the AMI Study. PLoS ONE.

[25] Rietman M.L., van der A D.L., van Oostrom S.H., Picavet H.S.J., Dollé M.E.T., van Steeg H., Verschuren W.M.M., Spijkerman A.M.W. (2018). The Association Between BMI and Different Frailty Domains: A U-Shaped Curve?. J. Nutr. Health Aging.

[26] Easton J.F., Stephens C.R., Román-Sicilia H., Cesari M., Pérez-Zepeda M.U. (2018). Anthropometric Measurements and Mortality in Frail Older Adults. Exp. Gerontol..

[27] Krakauer N.Y., Krakauer J.C. (2020). Association of Body Shape Index (ABSI) with Hand Grip Strength. Int. J. Environ. Res. Public Health.

[28] Kim M., Lee Y., Kim E.-Y., Park Y. Mediating Effect of Waist: Height Ratio on the Association between BMI and Frailty: The Korean Frailty and Aging Cohort Study. https://www.cambridge.org/core/journals/british-journal-of-nutrition/article/mediating-effect-of-waistheight-ratio-on-the-association-between-bmi-and-frailty-the-korean-frailty-and-aging-cohort-study/CCF95088B753D1ED366C8C436D963F3F.

[29] Coelho-Júnior H., Rodrigues B., Uchida M., Marzetti E. (2018). Low Protein Intake Is Associated with Frailty in Older Adults: A Systematic Review and Meta-Analysis of Observational Studies. Nutrients.

[30] Sandoval-Insausti H., Pérez-Tasigchana R.F., López-García E., García-Esquinas E., Rodríguez-Artalejo F., Guallar-Castillón P. (2016). Macronutrients Intake and Incident Frailty in Older Adults: A Prospective Cohort Study. J. Gerontol. Ser. A.

[31] Nanri H., Yamada Y., Yoshida T., Okabe Y., Nozawa Y., Itoi A., Yoshimura E., Watanabe Y., Yamaguchi M., Yokoyama K. (2018). Sex Difference in the Association Between Protein Intake and Frailty: Assessed Using the Kihon Checklist Indexes Among Older Adults. J. Am. Med. Dir. Assoc..

[32] Rahi B., Colombet Z., Gonzalez-Colaço Harmand M., Dartigues J.-F., Boirie Y., Letenneur L., Feart C. (2016). Higher Protein but Not Energy Intake Is Associated With a Lower Prevalence of Frailty Among Community-Dwelling Older Adults in the French Three-City Cohort. J. Am. Med. Dir. Assoc..

[33] Rashidi Pour Fard N., Amirabdollahian F., Haghighatdoost F. (2019). Dietary Patterns and Frailty: A Systematic Review and Meta-Analysis. Nutr. Rev..

[34] Kojima G., Avgerinou C., Iliffe S., Walters K. (2018). Adherence to Mediterranean Diet Reduces Incident Frailty Risk: Systematic Review and Meta-Analysis. J. Am. Geriatr. Soc..

[35] Bollwein J., Diekmann R., Kaiser M.J., Bauer J.M., Uter W., Sieber C.C., Volkert D. (2013). Dietary Quality Is Related to Frailty in Community-Dwelling Older Adults. J. Gerontol. Ser. A Biol. Sci. Med. Sci..

[36] Ntanasi E., Yannakoulia M., Kosmidis M.-H., Anastasiou C.A., Dardiotis E., Hadjigeorgiou G., Sakka P., Scarmeas N. (2018). Adherence to Mediterranean Diet and Frailty. J. Am. Med. Dir. Assoc..

[37] Machón M., Mateo-Abad M., Vrotsou K., Zupiria X., Güell C., Rico L., Vergara I. (2018). Dietary Patterns and Their Relationship with Frailty in Functionally Independent Older Adults. Nutrients.

[38] Parsons T.J., Papachristou E., Atkins J.L., Papacosta O., Ash S., Lennon L.T., Whincup P.H., Ramsay S.E., Wannamethee S.G. (2019). Physical Frailty in Older Men: Prospective Associations with Diet Quality and Patterns. Age Ageing.

[39] de Haas S.C.M., de Jonge E.A.L., Voortman T., Graaff J.S., Franco O.H., Ikram M.A., Rivadeneira F., Kiefte-de Jong J.C., Schoufour J.D. (2018). Dietary Patterns and Changes in Frailty Status: The Rotterdam Study. Eur. J. Nutr..

[40] Pilleron S., Ajana S., Jutand M.-A., Helmer C., Dartigues J.-F., Samieri C., Féart C. (2017). Dietary Patterns and 12-Year Risk of Frailty: Results From the Three-City Bordeaux Study. J. Am. Med. Dir. Assoc..

[41] Ghosh T.S., Rampelli S., Jeffery I.B., Santoro A., Neto M., Capri M., Giampieri E., Jennings A., Candela M., Turroni S. (2020). Mediterranean Diet Intervention Alters the Gut Microbiome in Older People Reducing Frailty and Improving Health Status: The NU-AGE 1-Year Dietary Intervention across Five European Countries. Gut.

[42] Siriwardhana D.D., Hardoon S., Rait G., Weerasinghe M.C., Walters K.R. (2018). Prevalence of Frailty and Prefrailty among Community-Dwelling Older Adults in Low-Income and Middle-Income Countries: A Systematic Review and Meta-Analysis. BMJ Open.

[43] El Zoghbi M., Boulos C., Awada S., Rachidi S., All Hajje A., Bawab W., Saleh N., Salameh P. (2013). Prevalence of Malnutrition and Its Correlates in Older Adults Living in Long Stay Institutions Situated in Beirut, Lebanon. J. Res. Health Sci..

[44] El-Hayeck R., Baddoura R., Wehbé A., Bassil N., Koussa S., Khaled K., Richa S., Khoury R., Alameddine A., Sellal F. (2019). An Arabic Version of the Mini-Mental State Examination for the Lebanese Population: Reliability, Validity, and Normative Data. J. Alzheimer’s Dis..

[45] Living Conditions of Households 2007 | UNDP in Lebanon. https://www.lb.undp.org/content/lebanon/en/home/library/poverty/living-conditions-of-households-2007.html.

[46] Maillet D., Matharan F., Clésiau H., Bailon O., Peres K., Amieva H., Belin C. (2016). TNI-93: A New Memory Test for Dementia Detection in Illiterate and Low-Educated Patients. Arch. Clin. Neuropsychol. Off. J. Natl. Acad. Neuropsychol..

[47] Morley J.E., Malmstrom T.K., Miller D.K. (2012). A Simple Frailty Questionnaire (FRAIL) Predicts Outcomes in Middle Aged African Americans. J. Nutr. Health Aging.

[48] Folstein M.F., Folstein S.E., McHugh P.R. (1975). “Mini-Mental State”: A Practical Method for Grading the Cognitive State of Patients for the Clinician. J. Psychiatr. Res..

[49] Lipschitz D.A. (1994). Screening for Nutritional Status in the Elderly. Prim. Care.

[50] Ambagtsheer R., Visvanathan R., Cesari M., Yu S., Archibald M., Schultz T., Karnon J., Kitson A., Beilby J. (2017). Feasibility, Acceptability and Diagnostic Test Accuracy of Frailty Screening Instruments in Community-Dwelling Older People within the Australian General Practice Setting: A Study Protocol for a Cross-Sectional Study. BMJ Open.

[51] Topolski T.D., LoGerfo J., Patrick D.L., Williams B., Patrick M.M.B. (2006). The Rapid Assessment of Physical Activity (RAPA) Among Older Adults. Prev. Chronic Dis..

[52] Guigoz Y. (2006). The Mini Nutritional Assessment (MNA) review of the literature—What does it tell us?. J. Nutr. Health Aging.

[53] U.S. Department of Health and Human Services and U.S. Department of Agriculture 2015–2020 Dietary Guidelines for Americans. 8th Edition. December 2015. http://health.gov/dietaryguidelines/2015/guidelines/.

[54] Gray W.K., Richardson J., McGuire J., Dewhurst F., Elder V., Weeks J., Walker R.W., Dotchin C.L. (2016). Frailty Screening in Low- and Middle-Income Countries: A Systematic Review. J. Am. Geriatr. Soc..

[55] Sibai A.M., Sen K., Baydoun M., Saxena P. (2004). Population Ageing in Lebanon: Current Status, Future Prospects and Implications for Policy. Bull. World Health Organ..

[56] United Nations, Department of Economic and Social Affairs, Population Division (2019). World Population Prospects 2019, Volume II: Demographic Profiles (ST/ESA/SER.A/427).

[57] Hoogendijk E.O., van Hout H.P.J., Heymans M.W., van der Horst H.E., Frijters D.H.M., Broese van Groenou M.I., Deeg D.J.H., Huisman M. (2014). Explaining the Association between Educational Level and Frailty in Older Adults: Results from a 13-Year Longitudinal Study in the Netherlands. Ann. Epidemiol..

[58] Veronese N., Stubbs B., Noale M., Solmi M., Pilotto A., Vaona A., Demurtas J., Mueller C., Huntley J., Crepaldi G. (2018). Polypharmacy Is Associated With Higher Frailty Risk in Older People: An 8-Year Longitudinal Cohort Study. J. Am. Med. Dir. Assoc..

[59] Hakeem F.F., Bernabé E., Sabbah W. (2019). Association between Oral Health and Frailty: A Systematic Review of Longitudinal Studies. Gerodontology.

[60] Ensrud K.E., Blackwell T.L., Redline S., Ancoli-Israel S., Paudel M.L., Cawthon P.M., Dam T.-T.L., Barrett-Connor E., Leung P.C., Stone K.L. (2009). Sleep Disturbances and Frailty Status in Older Community-Dwelling Men. J. Am. Geriatr. Soc..

[61] Kamil R.J., Li L., Lin F.R. (2014). Association of Hearing Impairment and Frailty in Older Adults. J. Am. Geriatr. Soc..

[62] Swenor B.K., Lee M.J., Tian J., Varadaraj V., Bandeen-Roche K. (2020). Visual Impairment and Frailty: Examining an Understudied Relationship. J. Gerontol. A Biol. Sci. Med. Sci..

[63] García-Esquinas E., José García-García F., León-Muñoz L.M., Carnicero J.A., Guallar-Castillón P., Gonzalez-Colaço Harmand M., López-García E., Alonso-Bouzón C., Rodríguez-Mañas L., Rodríguez-Artalejo F. (2015). Obesity, Fat Distribution, and Risk of Frailty in Two Population-Based Cohorts of Older Adults in Spain. Obesity.

[64] Bollwein J., Volkert D., Diekmann R., Kaiser M.J., Uter W., Vidal K., Sieber C.C., Bauer J.M. (2013). Nutritional Status According to the Mini Nutritional Assessment (MNA®) and Frailty in Community Dwelling Older Persons: A Close Relationship. J. Nutr. Health Aging.

[65] Chang S.-F. (2017). Frailty Is a Major Related Factor for at Risk of Malnutrition in Community-Dwelling Older Adults: Frail Assessment for Nutrition. J. Nurs. Scholarsh..

[66] Laclaustra M., Rodriguez-Artalejo F., Guallar-Castillon P., Banegas J.R., Graciani A., Garcia-Esquinas E., Ordovas J., Lopez-Garcia E. (2018). Prospective Association between Added Sugars and Frailty in Older Adults. Am. J. Clin. Nutr..

[67] Struijk E.A., Rodríguez-Artalejo F., Fung T.T., Willett W.C., Hu F.B., Lopez-Garcia E. (2020). Sweetened Beverages and Risk of Frailty among Older Women in the Nurses’ Health Study: A Cohort Study. PLoS Med..

[68] Feart C. (2019). Nutrition and Frailty: Current Knowledge. Prog. Neuro-Psychopharmacol. Biol. Psychiatry.

[69] Barrea L., Muscogiuri G., Di Somma C., Tramontano G., De Luca V., Illario M., Colao A., Savastano S. (2019). Association between Mediterranean Diet and Hand Grip Strength in Older Adult Women. Clin. Nutr..

[70] Fung T.T., Struijk E.A., Rodriguez-Artalejo F., Willett W.C., Lopez-Garcia E. (2020). Fruit and Vegetable Intake and Risk of Frailty in Women 60 Years Old or Older. Am. J. Clin. Nutr..

[71] Kojima G., Avgerinou C., Iliffe S., Jivraj S., Sekiguchi K., Walters K. (2018). Fruit and Vegetable Consumption and Frailty: A Systematic Review. J. Nutr. Health Aging.

[72] Garcia-Esquinas E., Rahi B., Peres K., Colpo M., Dartigues J.-F., Bandinelli S., Feart C., Rodriguez-Artalejo F. (2016). Consumption of Fruit and Vegetables and Risk of Frailty: A Dose-Response Analysis of 3 Prospective Cohorts of Community-Dwelling Older Adults. Am. J. Clin. Nutr..

