# Supplementary material for: Dietary Patterns and Risk Factors of Frailty in Lebanese Older Adults
Source: Nutrients. 2021 Jun 25;13(7):2188. doi: 10.3390/nu13072188 (PMC8308408; doi:10.3390/nu13072188)
Supplement: Supplementary file 1 [file nutrients-13-02188-s001.zip › nutrients-1243196-supplementary.pdf]

Supplementary Material

Table S1. Food groups and food items included in the cluster analysis.

Food group	Food items included	Portion size used
**Refined flour products**	All types of bread including Lebanese bread, crisp bread, croissant, "Mankouche" (Lebanese dough-based product)	1 oz of bread or ½ cup cooked cereals
**Whole cereals and products**	Whole wheat, whole wheat bread, oat breads and bulgur	
**Rice**	White rice	
**Pizza and pasta**	Bread equivalent of pizza, pasta	
**Corn and breakfast cereals**	Breakfast cereals and corn	
**Potato**	Potatoes and potato products	½ cup vegetable
**Raw vegetables**	Raw green leafy vegetables, tomatoes, cucumbers, mushrooms, sprouts and mixed salad/vegetables	
**Cooked vegetables**	Green beans, cooked cruciferous, zucchini, eggplants, carrots, pumpkins, bell peppers, sweet potatoes, green peas, onions, garlic	
**Fruits**	Fresh and dried fruits, fresh and bottled fruit juice	Usual portion size
**Legumes**	Lentils, chickpeas and beans, except soya	1 oz of meat or equivalent
**Meat & poultry**	Beef, veal, lamb/mutton, goat, poultry, organ meat, processed meat from red meat or poultry (e.g. Ham, sausages)	
**Eggs**	Eggs boiled or fried	
**Fish and shellfish**	Fresh and canned fish, fish products and seafood.	
**Vegetable oils**	Vegetable oils including corn, peanut, sunflower, and canola oils, and mayonnaise	1 teaspoon equivalent of fat
**Olive, seeds and oleaginous fruits**	Olives, olive oil, sesame paste ("Tahini") avocado, nuts	
**Processed and saturated fats**	Margarine, ghee and butter	
**Milk & dairy products**	Whole, light, or skimmed liquid milk, processed milk (condensed, dried), dairy dishes, cheeses	1 cup of milk equivalent
**Low fat sweets**	Wafers, "Meghle", gelatin-based dessert, milk-based puddings	Usual serving size
**High fat sweets**	Oriental sweets and baklava, cakes and desserts, chocolate	
**Sugars and jams**	Sugar and confectionery sugar, jam, marmalade, honey, "Halawa", soft drinks.	Teaspoon of sugar

Table S2. Consumption of predefined food categories according to dietary patterns for men and women separately. N=352.

	**Men(N=176)**				**Women (N=176)**			
	**WDP**	**HI-MED DP**	**MOD-MED DP**	**P**	**WDP**	**HI-MED DP**	**MOD-MED DP**	**P**
**Sample**	(N=29)	(N=21)	(N=126)		(N=13)	(N=60)	(N=103)	
**Age(years)**	70 (66-81)	79 (71-84)	73 (68-79)	0.183	75 (72-82)	71 (67-77)	73 (66-78)	0.238
**Refined cereals products**	6.6 (4.3-8.6)	5.9 (3-7.7)	3.5 (0.9-5.7)	0.01	4 (2.7-4.8)	1.5 (0.6-4.2)	3.01 (1.3-4.5)	0.002
**Whole breads & cereals (including burghul)**	0.4 (0.1-1.1)	0.7 (0.3-2.3)	1.06 (0.3-2.7)	0.07	1 (0.1-2.3)	2.05 (0.4-3.02)	0.5 (0.2-2.03)	0.004
**Potato**	0.6 (0.3-0.9)	0.4 (0.14-0.6)	0.3 (0.1-0.6)	0.16	0.3 (0.07-0.4)	0.2 (0.09-0.4)	0.2 (0.07-0.3)	0.305
**Vegetables**	2.8 (2.1-3.5)	4.3 (3-5.4)	3 (2.2-4.4)	0.13	3.3 (1.3-4.7)	3.6 (2.5-5.2)	2.8 (2-4.9)	0.071
**Fruits**	1.9(1.1-2.9)	3.01 (2.5-3.9)	2.1 (1.2-3.01)	0.01	2.4 (1.5-2.7)	2 (1.4-3.3)	1.8 (1.4-2.6)	0.099
**Legumes**	0.4 (0.2-0.9)	0.9 (0.6-1.15)	0.3 (0.13-0.6)	0.03	0.3 (0.2-0.6)	0.6 (0.2-0.9)	0.3 (0.07-0.6)	0.129
**Meat & Poultry**	2.6 (1.7-4.1)	3 (2.5-3.7)	2.1 (1.03-3.1)	0.01	1.6 (1.4-2.3)	1.9 (1.4-2.5)	1.8 (1.3-2.7)	0.445
**Eggs**	0.6 (0.3-0.9)	0.4 (0.3-0.7)	0.3 (0.1-0.6)	0.06	0.3 (0.1-0.9)	0.3 (0.1-0.5)	0.3 (0.07-0.5)	0.502
**Fish & shellfish**	0.4 (0.1-0.8)	0.5 (0.2-1.2)	0.4 (0.2-0.9)	0.95	0.4 (0.3-0.5)	0.4 (0.2-0.8)	0.2 (0.1-0.6)	0.071
**Milk and Dairy products**	1.8 (1.2-2.2)	2.4 (1.1-3.06)	1.3 (0.8-1.9)	0.03	2.3 (1-3.3)	1.6 (0.99-2.07)	1.45 (0.9-2.2)	0.061
**Vegetable oils**	2 (0.7-4)	3 (1-6)	3 (1.8-3)	0.17	3 (2-3)	3 (1.5-3.2)	3 (2-3.4)	0.829
**Olive, seeds and oleaginous fruits**	3.4 (3-5.03)	12.4 (10.7-15.4)	5 (3.4-6.8)	<0.001	4.4 (3.4-6.4)	9 (7.3-11.7)	3.8 (3-4.8)	<0.001
**Processed and saturated fats**	0.14 (0-1.2)	0 (0-0.4)	0 (0-0.2)	0.05	0 (0-0.6)	0 (0-0.14)	0 (0-0.14)	0.722
**Low Fat Sweets**	0.3 (0.04-1.4)	0.6 (0.13-1.1)	0.08 (0-0.3)	0.02	1.1 (0.5-2.3)	0.37 (0.08-1.9)	0.18 (0.02-0.5)	0.002
**High Fat Sweets**	0.2 (0.07-0.6)	0.3 (0.01-0.7)	0.06 (0-0.2)	0.01	0.2 (0.1-0.3)	0.12 (0-0.3)	0.07 (0-0.3)	0.074
**Sugars and jams**	7.1 (5.4-9.4)	4 (2.4-6.7)	1 (0.2-2.1)	<0.001	13 (9.7-18)	2.6 (0.8-4.05)	1.07 (0.14-2.1)	<0.001

Numeric variables are represented as median (interquartile range).

Abbreviations: WDP: Westernized dietary pattern; Hi-Med DP: high-intake Mediterranean dietary pattern; MOD-Med: moderate-intake Mediterranean dietary pattern.

Table S3. Nutritional characteristics of the dietary patterns.

	Total sample (N=352)				Men (N=176)				Women (N=176)			
	WDP	*HI-MED DP*	*MOD-MED DP*	***P-value***	*WDP*	*HI-MED DP*	*MOD-MED DP*	***P-value***	*WDP*	*HI-MED DP*	*MOD-MED DP*	***P-value***
**Calories/d**	2261 (1968-2838)	2028 (1652-2451)	1743 (1457-2122)	**<0.001**	2264 (2151-2864)	3017 (2033-3317)	1832 (1543-2277)	**<0.001**	1968 (1423-2625)	1886 (1582.5-2298)	1627 (1350-1853)	**0.001**
**KCal/kg BW**	31.2 (27.2-36.5)	28.2 (22.6-36.2)	24 (18.6-29)	**<0.001**	30.5 (27.2-34.9)	34.3 (28.2-42.8)	24.49 (18.95-28.55)	**<0.001**	32.64 (21.49-36.46)	26.49 (22.15-35.28)	23.7 (17.7-29.6)	**0.003**
**Protein (g)/d**	78.65 (70.4-98.7)	67.9 (53.7-89.6)	63.6 (47.8-78.2)	**<0.001**	80.2 (73.4-99.1)	94.1 (66.8-116)	68.3 (53.2-86.4)	**<0.001**	70.4 (46-83.2)	65.45 (51.3-82.55)	56.4 (44.9-68.7)	**0.08**
**Protein (g)/kg BW**	1.1 (0.9-1.3)	0.95 (0.7-1.3)	0.87 (0.6-1.08)	**<0.001**	1.1 (0.9-1.3)	1.2 (0.9-1.5)	0.91 (0.7-1.07)	**0.001**	1 (0.67-1.27)	0.9 (0.71-1.23)	0.8 (0.6-1.1)	**0.08**
**Carbohydrates (g)/d**	285 (229-331)	206 (166-261)	188 (155-241)	**<0.001**	294 (247-318)	344 (224-359)	203.5 (169-255)	**<0.001**	229 (163-335)	198 (157.5-213.5)	175 (139-215)	**0.022**
**Fat (g) /d**	102 (83.5-121)	106 (86.8-138)	81.1 (69.4-102)	**<0.001**	104 (87.8-119)	136 (90.2-162)	82.75 (71-108)	**<0.001**	90.7 (66.2-121)	101.5 (83.8-126)	79.3 (64.4-93.5)	**<0.001**
**MUFA (%TEI)**	16.81 (14.7-18.6)	21.8 (19.1-25.1)	18.5 (16.1-21.3)	**<0.001**	15.4 (14.5-18.1)	19 (16.8-22.6)	18.35 (16.1-20.8)	**0.005**	17.9 (16-18.6)	22.8 (19.9-25.4)	18.9 (16.2-21.6)	**<0.001**
**PUFA (%TEI)**	7.65 (6-9)	9 (7.3-10.6)	8.6 (7.5-10.4)	**<0.001**	6.8 (5.7-8.7)	9.3 (7.7-9.9)	8.5 (7.5-10.1)	**0.003**	8.1 (6.2-9.2)	8.95 (7.3-11.1)	8.7 (7.43-10.7)	**0.204**
**SAFA (%TEI)**	12 (10.5-13.6)	12.2 (11-13.9)	12.2 (10.3-14.1)	**0.66**	12.2 (9.9-14.1)	11.6 (10.6-12.7)	11.75 (9.9-13.6)	**0.838**	11.7 (11.1-12.9)	12.4 (11.15-14.5)	12.97 (11.1-14.6)	**0.438**
**ω3 FA (g/d)**	0.6455 (0.52-0.81)	0.96 (0.61-1.35)	0.62 (0.477-0.88)	**<0.001**	0.68 (0.57-0.96)	1 (0.7-1.68)	0.7105 (0.51-0.93)	**0.03**	0.63 (0.41-0.69)	0.95 (0.585-1.245)	0.54 (0.44-0.733)	**<0.001**
**ω6/ω3 ratio**	19.27 (12.5-24.34)	15.27 (11.11-20.87)	20.7 (13.26-24.57)	**0.002**	16.89 (11.05-24.34)	16.44 (12.5-19.2)	18.645 (12.34-23.94)	**0.408**	20.8 (13.3-24.34)	14.75 (10.86-22.19)	22.4 (15.5-25.2)	**0.001**
**Fibers (g/d)**	19.95 (16-23.6)	21.2 (17.3-28.2)	17.8 (13.7-23.9)	**<0.001**	20.5 (18.7-23.8)	30.3 (19.3-35.2)	19.2 (14.7-25.8)	**0.001**	18.2 (13.4-21.8)	20.35 (16.95-25.8)	16.4 (12.7-19.8)	**0.001**

Numeric variables are reported as median (interquartile range).

Abbreviations: WDP: Westernized dietary pattern; Hi-Med DP: high-intake Mediterranean dietary pattern; MOD-Med: moderate-intake Mediterranean dietary pattern; Kcal/d: Calories per day; Calories/kg BW: Calories per kg body weight; Protein/kg BW: Proteins per kg body weight; MUFA: mono-unsaturated fat acids; %TEI: percentage total energy intake; PUFA: poly-unsaturated fatty acids; SAFA: saturated fatty acids; FA: fatty acids; ω6/ω3 ratio: omega-6 polyunsaturated fatty acids/omega-3 polyunsaturated fatty acids ratio.
